# Case report: outcome of anlotinib treatment in breast cancer patient with brain metastases

**DOI:** 10.3389/fphar.2024.1381478

**Published:** 2024-08-19

**Authors:** Qiongwen Zhang, Xi Yan, Ting-Lun Tian, Xin Wu

**Affiliations:** ^1^ Department of Head and Neck Oncology, Department of Radiation Oncology, Cancer Center and State Key Laboratory of Biotherapy, West China Hospital of Sichuan University, Chengdu, China; ^2^ Breast Disease Center, Cancer Center, West China Hospital, Sichuan University, Chengdu, China

**Keywords:** anlotinib, breast cancer, brain metastases, case report, chemothearpy

## Abstract

Brain metastases (BM) represent a common and severe complication of breast cancer (BC), emerging in approximately 10%–16% of all BC patients. The prevalent approach for treating BC patients with BM encompasses a multimodal strategy, combining surgery, whole brain radiation therapy, and stereotactic radiosurgery. Yet, a concrete guideline for localized treatment strategies remains elusive, while systemic treatments like small-molecule-targeted therapy and immunotherapy are still in the clinical trial phase. This case study presents a significant clinical response to anlotinib treatment in a patient with estrogen receptor-negative, progesterone receptor-positive, and human epidermal growth factor receptor 2 (HER2)-positive breast cancer, complicated by BM. After the standard first-line treatment including albumin-bound paclitaxel, trastuzumab and pertuzumab, and a second-line treatment involving pyrotinib, capecitabine, and radiotherapy did not produce the desired results, the patient was then administered anlotinib in combination with pyrotinib and letrozole as a third-line treatment, which led to a partial response (PR). The findings suggest that anti-angiogenic therapy, specifically anlotinib, could be regarded as a promising therapeutic option for BC patients with BM.

## Introduction

Breast cancer (BC) stands as the most prevalent cancer among women and the leading cause of cancer-related mortality (Lukasiewicz et al., 2021; Giaquinto et al., 2022). Metastases from BC frequently target the lungs, liver, and bones (Riggio et al., 2021; Nathanson et al., 2022). However, the incidence of brain metastases (BM) is on the rise, attributed to more effective systemic treatments that improve disease management and extend patient survival. Concurrently, advancements in diagnostic imaging technology now facilitate the detection of even minute brain metastases (Wang et al., 2021). Current estimates suggest that up to 30% of patients with metastatic breast cancer eventually develop brain metastases (Lin et al., 2013; Martin et al., 2017; Wang et al., 2021). The occurrence of BM in BC patients is typically associated with a grim prognosis, compromised cognitive and sensory functions, and a significant decrease in the quality of life. Present therapeutic strategies for BC patients with BM include surgery, whole-brain radiation therapy (WBRT), stereotactic radiosurgery (SRS), chemotherapy, or a combination thereof (Bailleux et al., 2021). Despite these treatment modalities, current treatment of brain metastases in breast cancer patients is limited by the blood-brain barrier, tumor heterogeneity, limited treatment options, significant neurological impact, aggressive nature of metastases, and toxicity, all contributing to poor prognosis (Bailleux et al., 2021; Riggio et al., 2021). Consequently, there is an emergent need to identify novel therapeutic targets, augment treatment efficacy, and improve the prognosis of BC patients with BM.

Targeted therapy, involving HER2-targeted drugs like trastuzumab, pertuzumab, and pyrotinib, has significantly enhanced the prognosis of BC patients (Gabos et al., 2006; Li et al., 2017; Escrivá-de-Romaní et al., 2018; Collins et al., 2019). However, the efficacy of these drugs in treating BC patients with BM remains an active area of investigation (Li et al., 2019; Zhang et al., 2023). Anlotinib, a novel multi-targeted tyrosine kinase inhibitor (TKI), acts concurrently on various receptors, including vascular endothelial growth factor receptors (VEGFR1, VEGFR2/KDR, and VEGFR3), fibroblast growth factor receptors (FGFR1, FGFR2, FGFR3, and FGFR4), platelet-derived growth factor receptor (PDGFR), and stem cell factor receptor (c-kit) (Shen et al., 2018). Prior research has demonstrated that anlotinib can traverse the BBB and inhibit multiple signaling pathways, such as PI3K/AKT, MAPK/ERK, and RAF/MRK (Shen et al., 2018; Song et al., 2020; Li et al., 2023). By targeting multiple signaling pathways, anlotinib effectively disrupts the complex process of tumor angiogenesis, thereby displaying a direct inhibitory effect on tumor growth within the brain. Simultaneously, anlotinib has demonstrated a pronounced antiedema effect in various clinical studies, suggesting its potential to reduce the permeability of blood vessels associated with brain tumors. In this report, we detail the application of anlotinib in a 57-year-old woman with BM. She underwent third-line therapy involving anlotinib combined with pyrotinib and letrozole. Subsequent evaluations, including magnetic resonance imaging (MRI) and blood tests, indicated a partial response (PR). Therefore, this case suggests anlotinib may serve as a viable therapeutic option for BC patients with BM.

### Case description

**FIGURE 1:**
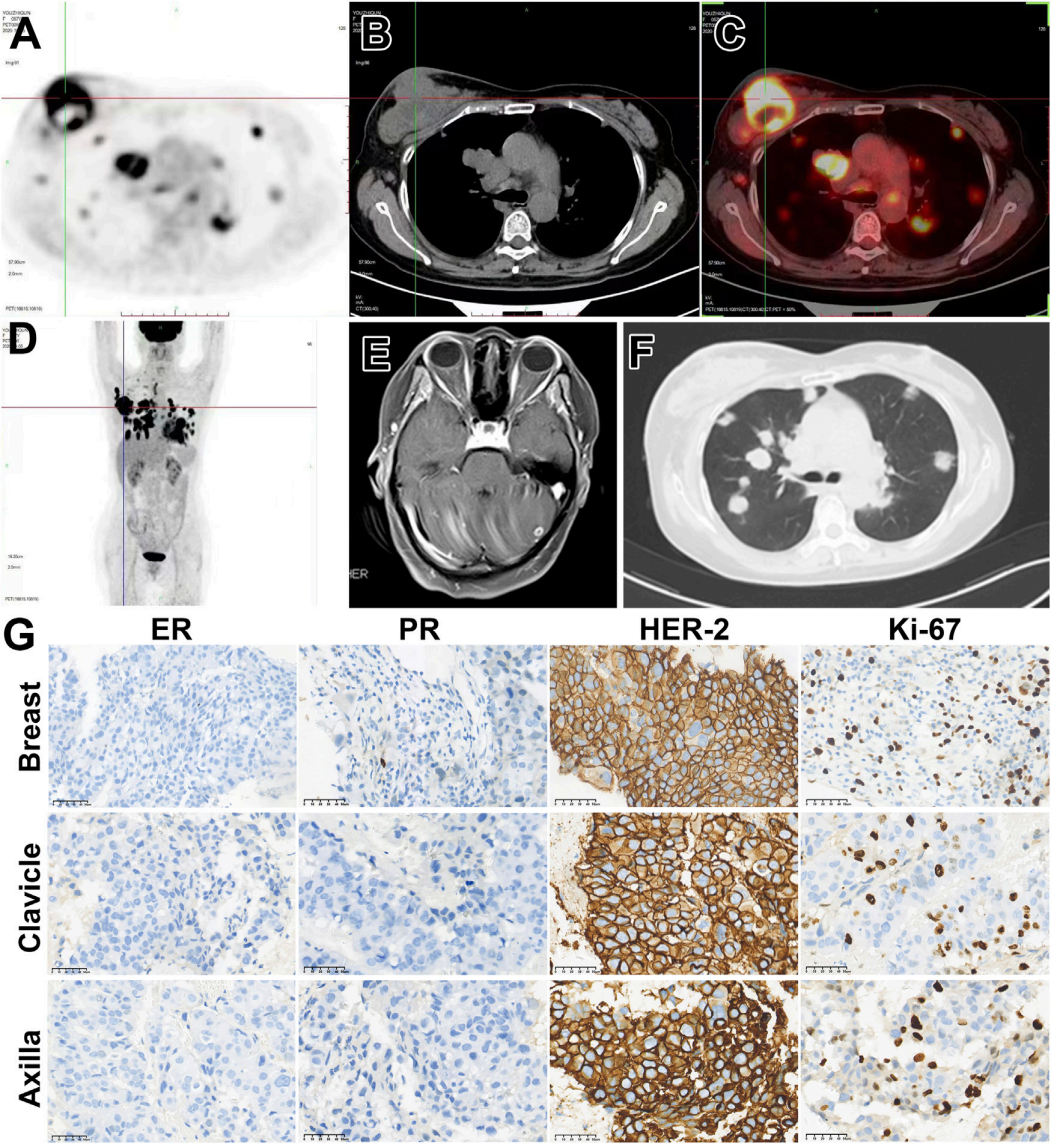
Patient’s imaging and diagnose before anti-tumor treatment. **(A–D)** ^18^F-FDG-PET/CT suggested a solid tumor (91 mm *× 51* mm *X* 78 mm) in the right breast with multiple metastases. **(E)** Enhanced MRI of the head indicates intracranial metastasis. **(F)** Chest CT shows multiple lung metastases. **(G)** Right breast biopsy indicates invasive carcinoma, ER (−), PR (+, approximately 20%), Her-2 (3+), Ki-67 35%–40%. Cancer cells are observed in the pathological biopsy of the lymph node above the right clavicle. Immunohistochemical staining shows ER (−), PR (+, approximately 10%), Her-2 (3+), Ki-67 35%–40%. Cancer cells are found in the pathological biopsy of the right axillary lymph node, ER (−), PR (−), Her-2 (3+), Ki-67 approximately 35%–40%.

A 57-year-old female patient was admitted to our hospital in December 2020 with a nearly year-long history of a progressively enlarging mass in the upper outer quadrant of her right breast. Ultrasonographic examination revealed a right BI-RADS 4c breast mass, measuring 91 × 51 × 78 mm, accompanied by swollen lymph nodes in the right axillary, supraclavicular, and infraclavicular regions. The pathology results from the biopsies obtained from the breast mass, as well as from the axillary and supraclavicular lymph nodes, all collectively confirmed the presence of invasive carcinoma characterized by estrogen receptor negative, progesterone receptor positive, and HER2 positive (ER^−^PR^+^HER2^+^), highlighting the critical role of molecular testing in guiding targeted treatment decisions, predicting therapy response, and facilitating personalized medicine. For example, HER2-positive cancers, may benefit from targeted therapies such as trastuzumab or pertuzumab, which specifically inhibit the HER2 protein. Other histological details include P63 positivity, CK5/6 positivity, and a Ki-67 positive rate of approximately 35%–40% in the region. Positron emission tomography/computed tomography (PET/CT) scanning disclosed metastases in the bilateral lungs, mediastinum, and bilateral hilar lymph nodes. A magnetic resonance imaging (MRI) scan indicated multiple intracranial occupancies, which, in conjunction with the patient’s medical history, suggested brain metastasis (Figure 1). The patient was classified as stage IV (cT4dN3M1).

**FIGURE 2:**
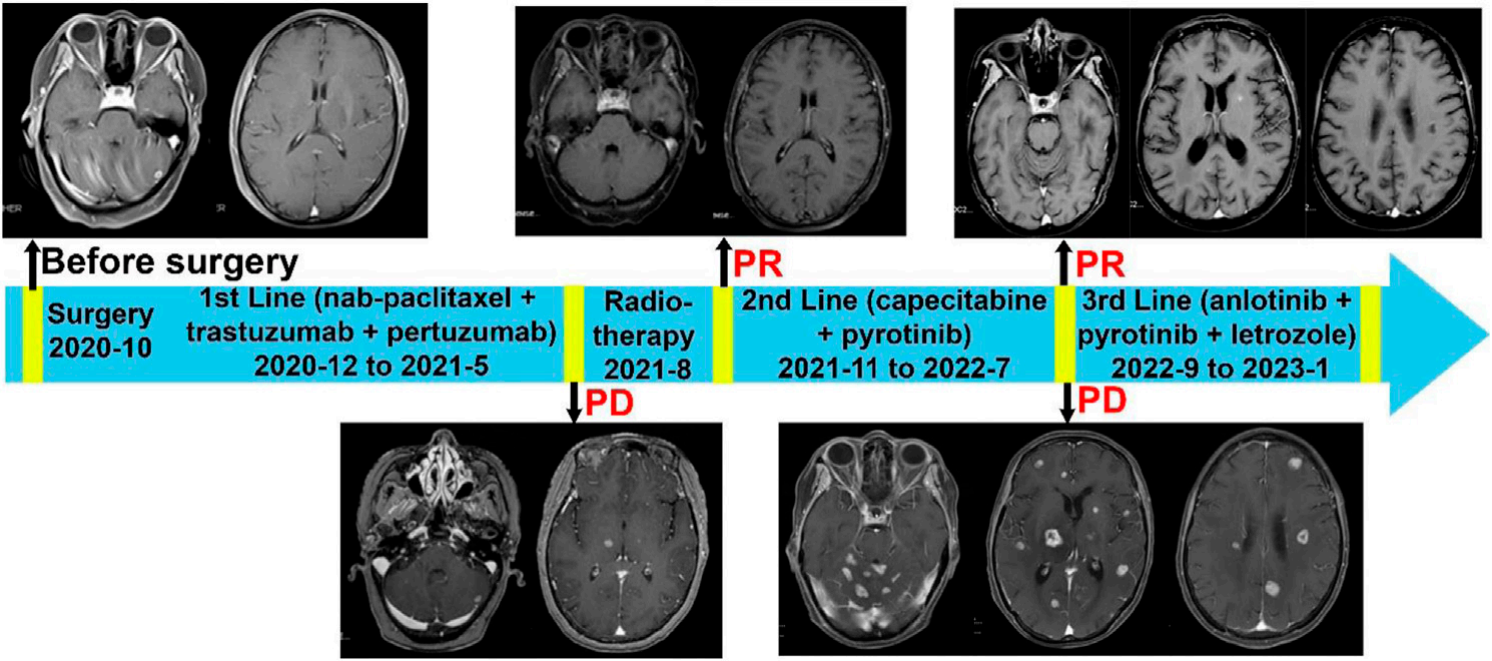
Complete treatment process of the patient since diagnosis. Brain MRIs of the patient were displayed. 1) Enhanced MRI of the head upon patient’s admission in December 2020 displays multiple intracranial metastatic tumors. 2) The enhanced MRI of the head from August 2021 shows that compared to the baseline scan, the size and number of intracranial metastatic foci have increased (prior to radiation therapy). 3) Follow-up enhanced MRI of the head after radiation therapy in September 2021 demonstrates a reduction in the number and size of the intracranial metastatic tumors. 4) Before the Anlotinib treatment, the enhanced MRI of the head in August 2022 shows that the size and number of multiple brain parenchyma metastatic tumors have increased compared to previous scans. 5) The follow-up enhanced MRI of the head in November 2022 shows a significant reduction in the number and size of metastatic tumor lesions compared to previous scans.

**FIGURE 3:**
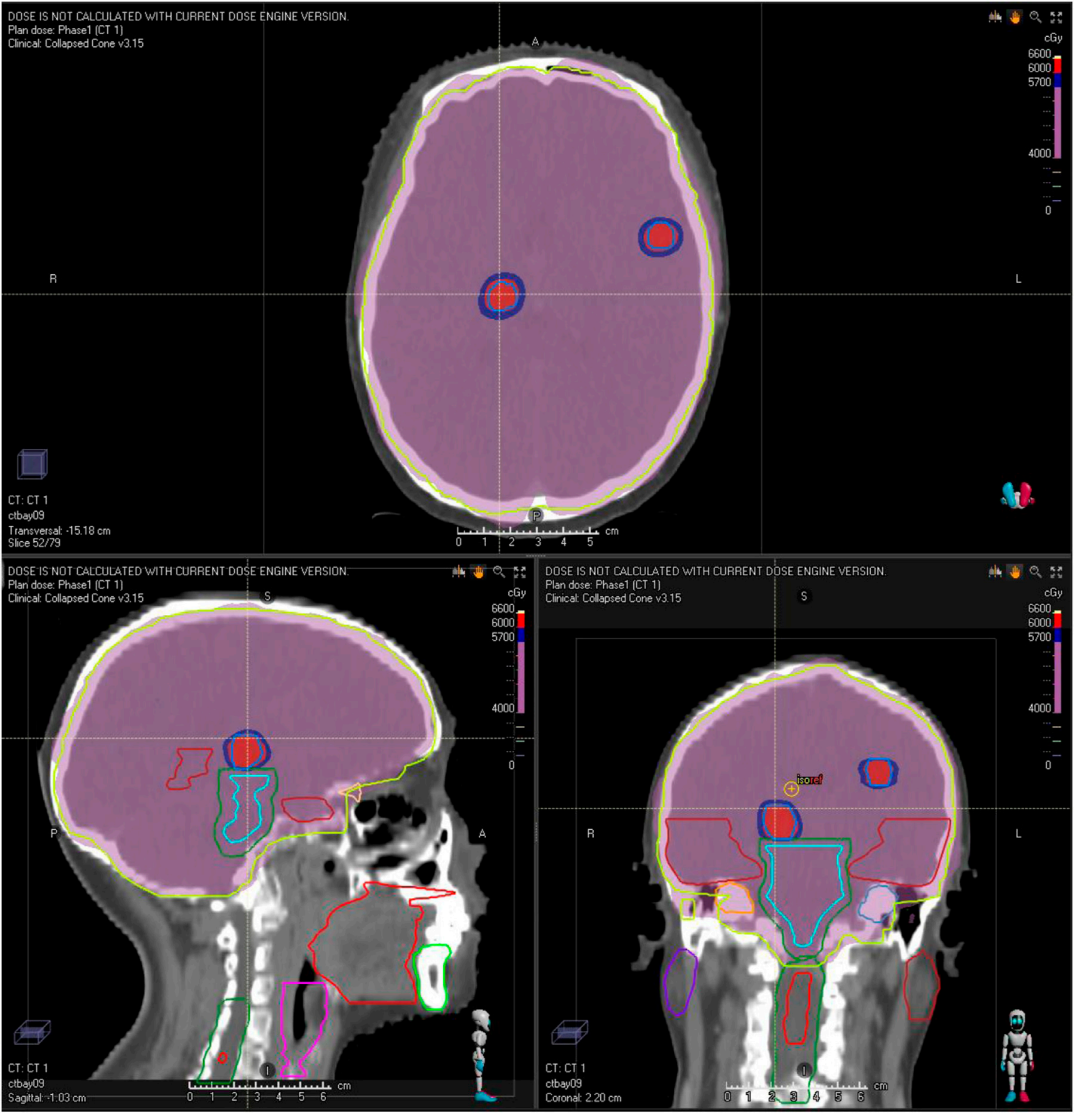
Illustration of the patient’s radiation treatment planning and dose distribution. The red and purple isodose color-wash indicate doses of 60 Gy (GTV) and 40 Gy (whole-brain), respectively.

The patient commenced first-line systemic chemotherapy in December 2020, following the albumin-bound paclitaxel, trastuzumab, and pertuzumab regimen (12 cycles each, Figure 2). An MRI review in June 2021 showed an increase in size and number of the patient’s intracranial metastases, with the therapeutic response evaluated as progressive disease (PD) (Figure 2). In June 2021, the patient began radiotherapy for the intracranial metastases, with a gross tumor volume (GTV) of 6000 cGy/20F (faction) and whole-brain irradiation of 4000 cGy/20F (Figure 3). Considering the patient’s multiple intracranial metastatic foci (more than 20), the patient was administered whole brain radiation therapy, while lesions with 0.8–1.5 cm in diameter were treated by local dose escalation radiation therapy. The patient tolerated the radiotherapy and other interventions well. A head MRI review in September 2021 indicated a reduction in the number and size of the patient’s intracranial metastases (Figure 2). The patient started oral administration of capecitabine 1.5 g twice daily and pyrotinib 400 mg once daily from November 2021. The patient’s general condition during treatment was good, with some gastrointestinal reactions such as diarrhea, nausea, and vomiting, but she was able to tolerate these. In the next follow-up, the patient experienced dizziness, and a head MRI review in August 2022 showed an increase in the size and number of the patient’s intracranial metastases, with the therapeutic response evaluated as PD (Figure 2).

**FIGURE 4:**
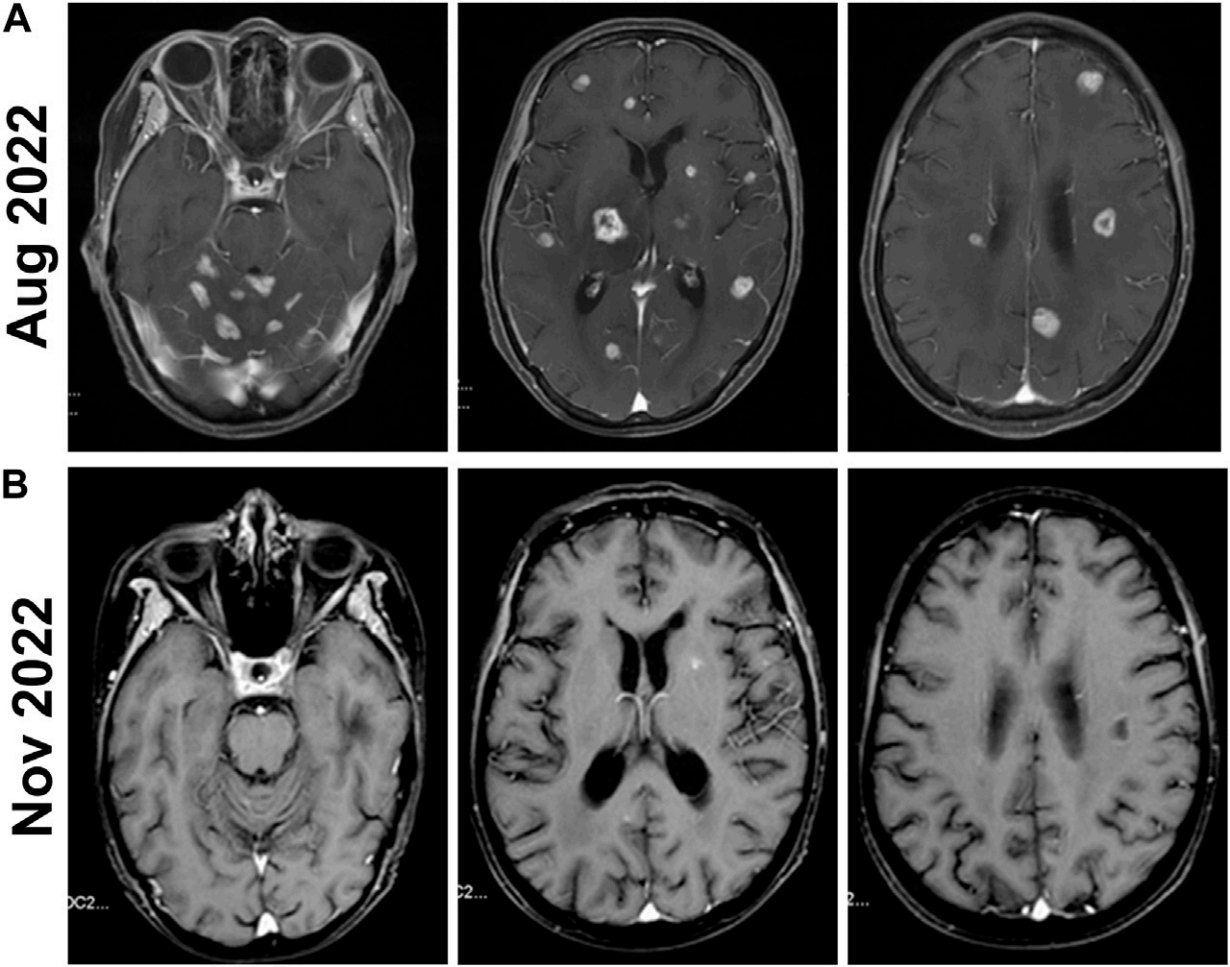
Brain MRIs of the patient before and after the Anlotinib treatment. **(A)** Before the Anlotinib treatment, the enhanced MRI of the head in August 2022 shows that the size and number of multiple brain parenchyma metastatic tumors have increased compared to previous scans. **(B)** The follow-up enhanced MRI of the head in November 2022 shows a significant reduction in the number and size of metastatic tumor lesions compared to previous scans.

Upon the PD in the second-line treatment with TKI, a change in the treatment plan was advisable but the option of switching to agents like T-DM1 or TDxd was not utilized due to budget constraints. Given the presence of multiple intracranial lesions and associated symptoms, anlotinib as an alternative TKI may offer enhanced effectiveness. In addition, an aromatase inhibitor (AI) was a plausible approach as endocrine therapy has not been previously administered. Considering the patient is ER-negative and PR-positive and the effectiveness of endocrine therapy was probably weak, only AI was initially added without the concurrent use of CDK4/6. Therefore, the third-line treatment plan was revised to include anlotinib 12 mg once daily, pyrotinib 400 mg once daily, and letrozole 2.5 mg once daily in September 2022. During this treatment phase, the patient experienced symptoms of diarrhea, which markedly improved following antidiarrheal treatment. The patient did not experience any adverse effects such as hypertension, bleeding, hand-foot syndrome, or thyroid function abnormalities. An MRI review in November showed a decrease in tumor size, indicating successful tumor control (Figure 4B).

## Discussion

BM originating from breast cancer ranks as the second most common source of BM, and the incidence of these frequently lethal lesions is currently increasing. Existing treatment modalities for BC patients with BM, including surgery, radiotherapy, chemotherapy, and targeted therapy, have yet to significantly alter the poor prognosis associated with BM. This underscores the pressing need to develop innovative, effective targeted therapies to combat these deadly tumors. In the case report presented, we observed a partial response in our patient during the third-line treatment regimen comprising anlotinib. This response was noted after the patient experienced disease progression after first-line and second-line treatments (Table 1).

**TABLE 1:** Patient demographics, laboratory results, and treatments from existing case reports of breast cancer with metastases.

First author (year)	Age, gender	HER-2 status	Metastasis site	First-line treatment	Second-line treatment	Third-line treatment
**This case report**	57, F	Positive	Brain	Albumin-bound paclitaxel, trastuzumab and pertuzumab	Pyrotinib, capecitabine and radiotherapy	Anlotinib pyrotinib and letrozole
Song and Sun (2022)	53, F	Positive	Liver	Epirubicin, cyclophosphamide, paclitaxel and adjuvant radiation	Docetaxel & Zercepac	Zercepac, pertuzumab, & albumin-bound paclitaxel
Huang et al. (2023)	42, F	Positive	Brain, bone and lymph node	Capecitabine plus pyrotinib	pyrotinib plus trastuzumab and capecitabine	Apatinib, trastuzumab and albumin-bound paclitaxel
Wu et al. (2022)	60, F	Positive	Brain and bone	Trastuzumab, pertuzumab, nab-paclitaxel and radiotherapy	trastuzumab, pyrotinib, & capecitabin	Disitamab Vedotin (RC48)
Yamashiro and Morii (2023)	60s, F	Negative	Brain	Exemestane treatment	palbociclib plus letrozole	Abemaciclib and letrozole

For BC patients with initial multifocal brain metastases exceeding ten lesions, the preferred treatment is whole-brain radiation therapy (WBRT), which can control tumor and improve survival (Gondi et al., 2022). For patients with a good performance status, an additional localized dose is beneficial (Tsao et al., 2018). In this case, this patient responded well to the initial course of treatment, achieving control for 1 year. However, subsequent multiple metastases precluded further radiotherapy, necessitating reliance on chemical therapy. Current medicine options on TKIs include T-DM1, TDxd, and breast 03 (Cortes et al., 2022), but these are financially inaccessible to the patient in developing countries, and finding alternative medications is crucial.

Anlotinib distinguishes itself from other TKIs with the following primary advantages. Firstly, it is a novel oral multi-targeted receptor tyrosine kinase inhibitor, exhibiting a broad spectrum of action in inhibiting tumor angiogenesis and growth. Its efficacy has been observed across a wide range of tumor types, including renal carcinoma, soft tissue sarcoma, medullary thyroid carcinoma, NSCLC, colorectal cancer, melanoma, thymic carcinoma, and adenoid cystic carcinoma (Shen et al., 2018; Syed, 2018; Chu et al., 2021; Hu et al., 2021; Xu et al., 2021). For example, A study by Xu et al. explored the use of anlotinib for patients with advanced cervical cancer as a second-line or later treatment. Among the forty-two patients, who had failed prior chemotherapy, the disease control rate was observed to be an impressive 94.9%, with a median progression-free survival (PFS) of 9.4 months (Xu et al., 2021). In addition, a phase II single-arm clinical trial investigating anlotinib as a later-line therapy for HER2-negative breast cancer reported a disease control rate of 80.8% and a median PFS of 5.22 months (Hu et al., 2021). Recent studies have also shown that anlotinib, in combination with other antitumor medications like eribulin (a novel, nontaxane microtubule dynamics inhibitor) or TQB2450 (a monoclonal antibody targeting PD-L1), demonstrates promising efficacy and manageable safety in previously treated advanced Triple-Negative Breast Cancer (TNBC) (Liu et al., 2023; Wang et al., 2023). Collectively, these outcomes emphasize anlotinib’s broad antitumor efficacy in a range of solid tumors. The mechanism underlying this broad-spectrum antitumor efficacy can be attributed to anlotinib’s suppressive action on the major angiogenesis-related pathway involving VEGF/PDGFRβ/FGFR2. Notably, there exists substantial interplay among the three primary signaling pathways and each pathway has the potential to engage in crosstalk via downstream signaling processes, particularly through the modulation of kinases such as ERK, Akt, and PI3K. Therefore, the simultaneous blockade of anlotinib targeting all three signaling pathways offers a more comprehensive and effective tumor suppression (Lin et al., 2018; Xie et al., 2018). Additionally, further research also showed that the anlotinib’s antiangiogenic effects altered the immune microenvironment (TME). Anlotinib reprograms the typical immunosuppressive TME into an immunostimulatory TME, thereby significantly inhibiting tumor growth (Liu et al., 2020; Su et al., 2022).

Secondly, anlotinib has the potential to cross the blood-brain barrier (BBB) and inhibit both tumor angiogenesis and tumor cell proliferation of brain tumors. The BBB presents a significant challenge in cancer treatment: it not only potentially restricts metastasizing cancer cells but also hinders the delivery of therapeutic drugs to brain tumors, which consequently diminishes intracranial response rates to treatments (Lockman et al., 2010). However, the capability of anlotinib in this context is promising. A retrospective study of anlotinib’s use in small cell lung cancer patients with brain metastases indicated its potent efficacy within the central nervous system (Zhou et al., 2022). Additionally, animal studies have shown that radiation therapy can enhance the ability of anlotinib to penetrate the intact BBB, particularly in the treatment of glioblastoma (Li et al., 2023). These findings, coupled with the observed partial response (PR) in our case study involving a BC patient with BM, underscore the potential of anlotinib to effectively cross the BBB.

Last but not least, anlotinib was able to attenuate cerebral edema associated with brain metastases and associated radiotherapy. Brain metastases and associated radiotherapy can escalate intracranial pressure and result in neurological symptoms, significantly impairing patient quality of life (Weissman, 1988; Ryken et al., 2010). Anlotinib, a multi-targeted agent, inhibits VEGFR, PDGFR, FGFR, and c-kit, all of which are cellular factors that foster angiogenesis and enhance vascular permeability. Multiple clinical studies and case reports have demonstrated that anlotinib effectively changed vascular permeability, thereby reducing cerebral edema [(Wang et al., 2020; Yang et al., 2021; Zhuang et al., 2021)]. In our case, there was a notable decrease in the edema index (EI) following anlotinib treatment (Figure 2). Concurrently, the patient reported a reduction in edema-related symptoms. This suggests that anlotinib’s efficacy in managing cerebral edema may be comparable to other anti-vascular therapies, such as bevacizumab, positioning it as a potential alternative treatment for edema in patients with brain metastases. Meanwhile, the adverse events associated with anlotinib, including hypothyroidism, hypertension, and hand-foot syndrome, were relatively infrequent, with incidence rates ranging from 33% to 4% (Syed, 2018; Chu et al., 2021; Hu et al., 2021). This aligns with our case, where the patient reported no significant adverse events, highlighting anlotinib’s minimal toxicity profile.

There are several limitations in this case. 1) The patient was under anlotinib treatment for a short duration of just over 2 months. Despite demonstrating significant therapeutic effectiveness, the patient did not continue treatment due to the COVID-19 pandemic and subsequently was lost to follow-up, hence long-term efficacy data could not be obtained. 2) During the patient’s use of anlotinib, pyrotinib and letrozole were also administered concurrently. The potential contributions of these two additional drugs to the therapeutic effect cannot be ruled out, although tumor progression was observed during the prior use of pyrotinib. 3) This case report only presents the therapeutic effect of anlotinib on a patient with estrogen receptor-negative (ER-), progesterone receptor-positive (PR+), and human epidermal growth factor receptor 2-positive (HER2+) brain metastases. The effectiveness of anlotinib on other breast cancer subtypes, especially the highly malignant triple-negative breast cancer, still needs further investigations.

In conclusion, this case suggests that anlotinib could achieve clinical benefits in HER2-positive BC patients with BM, even after failing multiple lines of therapy. Anlotinib emerges as a promising new treatment alternative for patients battling advanced intracranial metastases resulting from breast cancer. These preliminary observations warrant further investigation through additional clinical trials to validate anlotinib’s efficacy.

## Data Availability

The original contributions presented in the study are included in the article/Supplementary Material, further inquiries can be directed to the corresponding author.
